# Skin biomechanical properties and leg volume in aging healthy adults

**DOI:** 10.1111/srt.13087

**Published:** 2021-08-29

**Authors:** Nyree Dunn, Jane Davies, Gina Dolan, E. Mark Williams

**Affiliations:** ^1^ Faculty of Life Sciences and Education University of South Wales Pontypridd UK; ^2^ Huntleigh Diagnostics ARJO Cardiff UK

**Keywords:** elastance, leg volumetry, myo‐tonometry, skin biomechanics, tone

## Abstract

**Background:**

In adults ageing is accompanied by changes in limb volume and skin biomechanical properties.

**Objectives:**

To explore the relationship between body size, aging, skin biomechanics, and leg volume, *V*_Leg_ and to define predictive equations linking leg volume with these properties.

**Methods:**

Sixty‐two healthy adults (Age 18–80 years, M:F 45:55) were recruited. Anthropomorphic measures were recorded along with *V*_Leg_ (via circumferential method) and skin tone, stiffness, and elastance (via tonometry). Regression analysis was performed to define relationship between the measured parameters.

**Results:**

In healthy adults bilateral *V*_Leg_ were the same regardless of leg or sex, 5791 ± 1363 for females and 6151 ± 1203 mls for males. *V*_Leg_ was positively correlated to body weight, where *V*_Leg_ (mls) = 1058 + 69 × Wt (kg) for females and *V*_Leg_ (mls) = 539 + 65 × Wt (Kg) of body weight for males. Skin surface biomechanical properties varied with sex, leg volume, and location on the leg with the malleolus exhibiting the stiffest surface.

**Conclusion:**

The study shows that anthropometric measures change with sex and leg size are multifactorial and body weight, sex, and skin condition as important determinant factors of leg volume.

## 1 INTRODUCTION

Often anthropomorphic studies of healthy adult legs measure limb muscle mass, adipose tissue content, and limb circumference but rarely address the issue of limb volume or the skin covering the limb. The ability of the leg to increase in volume is dependent on the skin's ability to stretch. In studies that have investigated limb volume various methods have been used and do not provide predictive equations related to body size or mass. The earliest measures of limb volume were achieved by simply measuring the water displaced following immersion of the leg or even the whole body in a water filled vessel.^1^ Later more sophisticated methods were developed, such as water‐displacement plethysmography, which was used to measure small changes in leg volume that are associated with altered blood supply to the limb.^2^ In terms of assessing limb muscle mass alone, the circumference of the leg muscle at set anatomical points proved sufficient to provide a good estimate. The evolution of ever more sophisticated technology now allows muscle mass (and limb volume) to be measured using electrical impedance, or with digital imaging technology such as X‐ray and Dual‐energy X‐ray absorptiometry (DEXA) scanning, with magnetic resonance imaging (MRI) and ultrasound scanning being the most recent techniques pioneered.^3^ In the healthy leg, bone and muscle contribute to the majority of the volume, but in some clinical conditions limb volume can be raised considerably, one such condition being lymphoedema. When lymph drainage is restricted, fluid remaining in the limbs expands limb volume, particularly in the lower limb between the ankle and knee and in the upper limb above the knee. The traditional method for measuring this enlargement has to been to use a series of limb circumferential measurements separated by 4 cm and using these dimensions to calculate limb volume using a truncated cone model.^4^ A simple tape measure can be used to complete this measure, although modern surface scanning techniques typified by the perometer have been developed to provide a similar estimate.^5^, ^6^ While there are many studies using this technique in people with lymphoedema there is little comparative measures performed on healthy non‐oedematous legs.

Skin biomechanics such as its viscoelastic properties are rarely reported along with lower limb volume in healthy people. While biomechanical properties should be independent of volume, there may be some differences in legs with differing layers of adipose tissue immediately below the surface, which tends to develop in females with age. Furthermore, in lymphoedema, where fluid collects under the skin, it is likely that the skin viscoelastic properties may change with volume.^7^, ^8^

The aim of this study was to establish the anthropometric relationships between skin biomechanics and limb volume in healthy adult males and females of differing age. Further aims were to derive predictive equations of the relationship between volume and body properties and to compare limb properties between the distal and proximal leg.

## 2 MATERIALS AND METHODS

### 2.1 Participants

Adult participants (*n* = 62, aged 18–80 years, M:F, 45:55%) were recruited from two sites in Wales, the University of South Wales, Pontypridd and from Huntleigh Healthcare Ltd, Cardiff, UK. The study was ethically approved by the Faculty of Life Sciences and Education Ethics sub‐Committee, University of South Wales, UK. All participants who met the inclusion criteria provided informed written consent (Table 1).

**TABLE 1:** Study Inclusion and Exclusion Criteria

**Inclusion Criteria**:
Age 18 or over. Able to provide written consent
**Exclusion Criteria**:
Any signs or confirmed diagnosis of lymphoedema (ISL stage II or III). Severe skin problems, lower limb ulcers or wounds. Non‐pitting chronic oedema. Known or suspected DVT. Pulmonary embolism or oedema, Thrombophlebitis, Acute inflammation of the skin (erysipelas and cellulitis). Ischaemic vascular disease. Active metastatic diseases affecting the oedematous region. Oedema at the root of extremity or truncal oedema.

### 2.2 Measurement procedures

Each participant while wearing light clothing and no footwear were weighed (Seca 877 Floor scales for mobile use, Class III) followed by a measure of their height using a stadiometer (Seca Leicester Portable). Later their body mass index, BMI, was calculated accordingly, BMI = kg/m^2^. Additional measures obtained from 35 participants were hip and waist circumference and leg length, all made using a tape measure with the participant standing upright.

**FIGURE 1:**
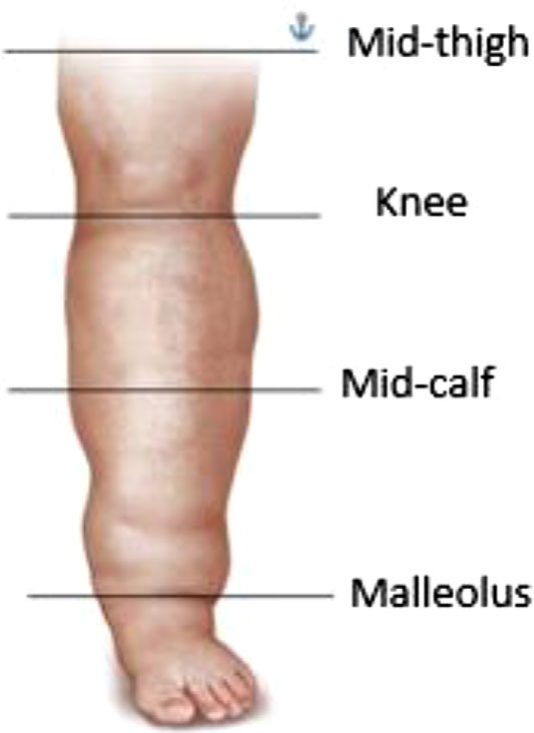
Anatomical points measured

All participants while seated underwent bilateral leg volume assessment using the standard tape measure method used in lymphoedema studies.^5^ Measurements were made utilizing a metric nonstretch tape measure (Medi Ltd, Germany). Leg circumference was measured at several points starting just above the malleolus and then at 4 cm intervals ascending the leg to the knee (the distal leg), and then from above the knee up to the upper thigh (the proximal leg).^5^, ^6^ A portable hand‐held myotonometer (Myoton AS, Tallinn, Estonia) was used to quantitatively assess the skins mechanical and viscoelastic properties.^9^ This palpation device uses a preloaded probe to briefly compress the skin and then follows the decay of the natural oscillatory response to the applied load.^10^ The myotonometer provides three measures, two mechanical properties, tone (Hz) which reflects the oscillation frequency characteristics of the skin in its passive state, and stiffness (N/m), or dynamic stiffness a measure of the skins resistance to the applied external force (load) deforming the skin from its initial shape. An increase in these parameters signifies an increase in the tone and stiffness of the skin, an undesirable characteristic of skin. ^11^ The third property is elasticity a measure of the skin's ability to recover its initial shape after the removal of an applied load, the device records the skin's speed of movement (acceleration) at the point of maximum depression, this parameter is termed the logarithmic decrement, and the measure is inversely related to elasticity, thus a decrease in the logarithmic decrement parameter reflects an increase in elasticity.^12^ The myotonometer was applied to four anatomical sites on each leg; immediately above the malleolus, the mid‐calf (akin to the distal portion of the leg), the knee, and finally the mid‐thigh, the proximal portion of the leg, resulting in eight measures per person (Figure 1).

### 2.3 Statistical analyses

Descriptive statistics were performed to show means and standard deviations in the participant population, leg volume and skin properties. Relationships between parameters were assessed using *t*‐tests and ANOVA. Regression procedures were used to define changes over ranges. Data were further stratified by leg side (left versus right) and sex (female versus male). A *p* value of < 0.05 was considered significantly different. Statistical analysis was performed using computer software (SigmaPlot, V14, Systat Software Inc, UK). Unless stated otherwise a single mean value from both left and right legs were used in the analysis.

## 3 RESULTS

### 3.1 Body composition

Females (*n* = 34) had a lower body weight (on average 16 kg less) and were smaller (on average 0.13 M less) than the male participants (*n* = 28) (Table 2). Ratio measurements such as the BMI and the Waist to Hip (W‐H) ratio were no different between the sexes (Table 2). The female leg length was shorter by a mean of 6 cm, while the leg volumes (the average of the left and right leg volume) did not differ significantly at around 6053 ± 1286 mls (Table 2). While the volume of the distal region was larger than the volume of the proximal region volume there were no sex differences (Table 2). There were no differences between the right leg volume (F:M 5987 ± 1436 ml: 6188 ± 1183 ml, *p *= 0.556) and left leg (F:M 5956 ± 1308 ml: 6114 ± 1227 ml, *p* = 0.629) volumes between the sexes.

**TABLE 2:** Participant anthropometric data

	Females (*n* = 34)	Males (*n* = 28)	All (*n* = 62)	*p*
Age (years)	45 (18–80)	39 (19–77)	45 (18–80)	0.308^#^
Weight (Kg)	71 (46–115)	87 (51–128)	78 (46–128)	<0.0001^#^
Height (M)	1.63 ± 0.06	1.76 ± 0.06	1.69 ± 0.08	<0.0001^*^
Body mass index	26 (18–38)	28 (18–37)	26 (18–38)	0.112^#^
Mean leg length (cm)	93 ± 1	99 ± 3	97 ± 4	0.017
Hip circumference (cm)	99 ± 7	107 ± 9	105 ± 9	0.165
Waist circumference (cm)	92 ± 8	102 ± 7	99 ± 8	0.065
Waist to hip ratio	0.93 ± 0.03	0.96 ± 0.08	0.95 0.07	0.599
Mean leg volume^a^ (mls)	5971 ± 1363	6151 ± 1203	6053 ± 1286	0.589
Distal Volume (mls)	3020 (2026–5050)	3281 (1958–4604)	3164 (1958–5050)	0.072^#^
Proximal Volume (mls)	2910 ± 739	2864 ± 755	2889 ± 741	0.808
Right leg volume (mls)^a^	5987 ± 1436	6188 ± 1183	6078 ± 1321	0.556
Left leg volume (mls)^a^	5956 ± 1308	6114 ± 1227	6028 ± 1264	0.629

^#^ Median with range shown.

^a^ Limb distal (lower leg, below the knee) and proximal (upper leg above the knee) volumes combined.

**FIGURE 2:**
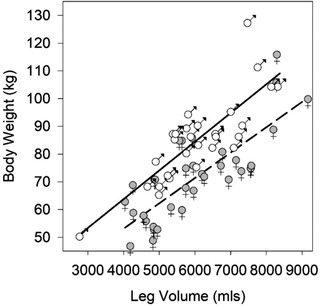
Relationship between body weight and leg volume in females (♀) and males (♂). A fitted linear regression line is shown for each plot, female (dashed line), *R* ^2^ = 0.631, Body weight (kg) = 16.59 + 9.14 × 10^−3^ × Leg volume (mls), male (solid line), *R* ^2^ = 0673, Body weight (kg) = 22.36 + 0.010 × Leg volume (mls)

The leg volume increased with body weight (Figure 2 and Table 3). In males there were also relationships between leg volume with age and height, with volume decreasing with age and increasing with height (Table 3). Forward stepwise regression analysis including weight, height, and age showed that in both sexes weight alone was sufficient to predict leg volume (*p* < 0.001).

**TABLE 3:** Correlation between total leg volume and body characteristics

	Sex	Linear regression analysis (*Y* = *a* + *bX*)
Age (years)	F	Volume (mls) = 5810 + 3.39 × Age (years), *R* ^2^ = 0.002, *p* = 0.813
	M	Volume (mls) = 7400 + 25.3 × Age (years), *R* ^2^ = 0.182, *p* = 0.027
Weight (kg)	F	Volume (mls) = 1058 + 69.05 × Weight (kg), *R* ^2^ = 0.631, *p* < 0.001
	M	Volume (mls) = 538.7 + 65.23 × Weight (kg), *R* ^2^ = 0.675, *p* < 0.001
Height (m)	F	Volume (mls) = −6078 + 78.16 × Height (m), *R* ^2^ = 0.101, *p* = 0.067
	M	Volume (mls) = −11017 + 97.4 × Height (m), *R* ^2^ = 0.197, *p* = 0.018

### 3.2 Skin mechanical properties

**FIGURE 3:**
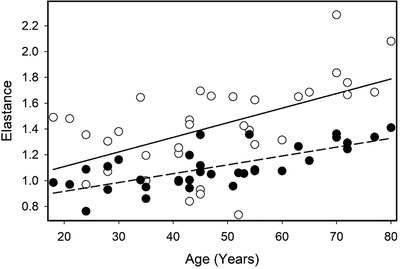
Relationship between skin elasticity (Log decrement) and age (years). Mean elasticity of the distal and knees sites (●, —–, *R* ^2^ = 0.52, Elasticity (Log decrement) = 0.78 + 6.58 × 10^−3^ × Age (years), and the proximal site (○ ^_,^ *R* ^2^ = 0.30, Elasticity (Log decrement) = 0.88 + 0.011 × Age)

The skins mechanical properties tone and stiffness measured at the four anatomical points ascending each leg were not related to the participant's age, weight, or height. The only exception being the relationship between body weight and skin stiffness at the knee (stiffness ((N/m) = 92.04 − 0.06 × weight, *p* = 0.02). Skin elasticity decreased with age at three anatomical sites, but no relationship existed between body weight or height (Table 4, Figure 3).

**TABLE 4:** Relationship between skin viscoelastic properties, anatomical sites, and sex

	Anatomical Zone			
Parameter	FM (*p*)	Distal (*p*)	Knee (*p*)	Proximal (*p*)
Tone (Hz)	F: 27.3 ± 4.4 M: 29.8 ± 4.2 0.03 All: 28.4 ± 4.5	19.0 ± 2.3^a^ 22.3 ± 2.1^a^ < 0.001 20.5 ± 2.7	16.3 ± 2.9^a^ 20.1 ± 4.2^a^ < 0.001 18.0 ± 4.0	14.0 ± 2.9^a^, ^b^ 18.6 ± 3.3^a^, ^b^ < 0.001 16.1 ± 3.8
Stiffness (N/m)	F: 692.6 ± 124.9 M: 743.9 ± 125.9 N.S All: 715.8 ± 126.9	398.5 ± 66.5^a^ 457.4 ± 66.8 < 0.001 425.1 ± 72.4	344.9 ± 100.8^a^ 448.6 ± 145.3 < 0.001 391.7 ± 132.5	272.4 ± 77.6^a^, ^b^ 354.5 ± 90.0 < 0.001 309.5 ± 92.4
Elastance	F: 1.09 ± 0.26 M: 0.95 ± 0.19 0.02 All: 1.03 ± 0.24	1.12 ± 0.19^a^ 0.90 ± 0.21^a^ < 0.001 1.02 ± 0.22	1.09 ± 0.18^a^ 0.99 ± 0.12^a^ 0.02 1.05 ± 0.16	1.42 ± 0.35^a^, ^b^ 1.42 ± 0.30 ^a^, ^b^ N.S 1.42 ± 0.32

The mean ± SD is shown. N.S: not significantly different, *p* > 0.05. The p value shown denotes a difference between female and male participants using a *t*‐test. Comparison between anatomical sites for each sex were made using a Kruskia‐Wallis one‐way ANOVA and a Post Hoc Tukey test if significance was found,

^a^ significantly different to the malleolus site,

^b^ significantly different to the distal site.

In males the three mechanical properties at the knee correlated with age, height, and weight in most comparisons (exceptions being elastance with weight and height) (Table 5).

**TABLE 5:** Male skin characteristics in relation to age, weight, and height

	Property and Linear regression characteristics
Age (years)	Tone (Hz) = 13.44 + 0.15 × Age (years), *R* ^2^ = 0.413, *p* < 0.001
	Stiffness (N/m) = 218 + 5.3 × Age (years), *R* ^2^ = 0.414, *p* < 0.001
	Elasticity = 0.86 + 0.003 × Age (years), *R* ^2^ = 0.200, *p* = 0.017
Weight (kg)	Tone (Hz) = 32.04 – 0.14 × Weight (kg), *R* ^2^ = 0.248, *p* = 0.007
	Stiffness (N/m) = 818.9 − 4.3 × Weight (kg), *R* ^2^ = 0.201, *p* = 0.017
	Elasticity = 0.94 − 0.0006 × Weight (kg), *R* ^2^ = 0.007, *p* = 0.678
Height (m)	Tone (Hz) = 80.9 − 0.345 × Height (m), *R* ^2^ = 0.200, *p *= 0.017
	Stiffness (N/m) = 2384 – 11.0 × Height (m), *R* ^2^ = 0.171, *p* = 0.029
	Elasticity = 1.69 – 0.004 × Height (m), *R* ^2^ = 0.03, *p* = 0.36

### 3.3 Relationship between limb volume and skin viscoelastic properties

**FIGURE 4:**
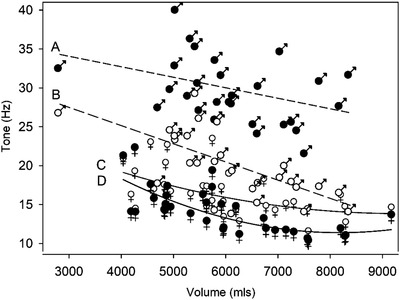
Relationship between skin tone (Hz) and leg volume (mls). Curve A (closed ♂) male malleolus skin tone (*R* ^2^ = 0.15, Tone (Hz) = 38.11+ −1.35 × Leg volume (mls)). Curve B (open ♂) male knee skin tone (*R* ^2^ = 0.45, Tone (Hz) = 34.52 + −2.34 × Leg volume (mls)). Curve C (open ♀) female knee skin tone (*R* ^2^= 0.30, Tone (Hz) = 30.32 + −3.55 × 10^−3^ × Leg volume + 1.91 × 10^−7^ × Leg volume^2^ (mls). Curve D (closed ♀) female proximal skin tone (*R* ^2^ = 0.52, Tone (Hz) = 37.99 + −6.51 × 10^−3^ × Volume (mls) + 3.98 × 10^−7^ × volume^2^ (mls)

**FIGURE 5:**
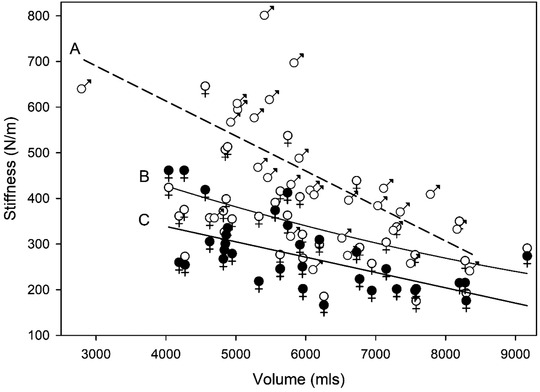
Relationship between skin stiffness (N/m) and leg volume (mls). Line A, male (♂) knee skin stiffness (*R* ^2^ = 0.40, Stiffness (N/m) = 920.2 + −0.77*x* Volume (mls). Curve B, female (open ♀) knee skin stiffness (*R* ^2^ = 0.27, Stiffness = 651.75 + −0.06X + 2.07 × 10^−6^ *X* ^2^). Line C female (closed ♀) proximal skin stiffness (*R* ^2^ = 0.35, *Y* = 472.88 + −0.034*X*)

In both sexes limb volume and skin biomechanics were correlated. With female limbs the tone and stiffness of the skin covering the knee and proximal sites decreased with increasing limb volume, *V_L_* (Figure 4 and 5). In males the skin tone of the knee and malleolus decreased with increasing limb volume (Figure 4) as did skin stiffness at the knee (Figure 5). Skin elastance was independent of limb volume in males and females at all four anatomical sites.

## 4 DISCUSSION

This is the first study to report the relationship between lower limb volume and skin biomechanical properties measured by the myotonometer. It is important in that it shows that these skin properties change with volume and that myotonometry is a useful tool in this assessment.

The study population is representative of the general UK population, matching the national mean heights and weight of 1.62 ± 0.14 and 1.76 ± 0.17 meters (mean and SE shown) and 72.1 ± 034 and 85.4 ± 0.41 kg for females and males.^13^ While females were smaller than the male participants, their measured leg volumes were the same (the combined proximal and distal regions); a positive correlation, with volume increasing with body size, was evident. Furthermore, the left and right leg volume were similar, suggestive of a symmetry between the limbs. The limb volumes measured in this study match those reported by other studies of healthy populations. Three studies ^14^, ^15^, ^16^, ^17^ reporting lower limb volumes of 2353, 2250, and 2826 mls, respectively in healthy adults compared to 2889 mls here (Table 2). A study of leg volume in elite athletes found that leg volumes increased with weight, in males from 12002 mls at 57–61 kg to 21400 mls at 125 kg in males and for females from 11 000 mls at 48–51 kg to 17 150 mls at 71–75 kg.^18^ The current study shows that regardless of sex, leg volume was positively related to body weight (Table 4), increasing by around 69 for females and 65 for males with every kg increase in body weight (Table 4). The increase in volume in this study is more likely due to additional adiposity rather than muscle (as in elite athletes) or fluid (as found in those with lymphoedema).^3^, ^19^ In males, the increase in volume was related to height, with those of greater height having the larger volumes; whether this was due to longer legs could not be determined. A loss of male leg volume with age is most likely due to general loss of muscle mass; this condition is known as sarcopenia and is characterized by the degenerative loss of skeletal muscle mass, quality, and strength in older persons.^20^

The viscoelastic properties of skin differs between anatomical sites and the sexes (Table 4). The skin at the malleolus had a higher tone, stiffness, and elastance than the other three sites, in both sexes. Female skin exhibited a lower tone, stiffness, and elastance than male skin. Other studies using the myotonometer have shown similar differences with anatomical sites, such as the shin and the calf, being 657 and 380 N/M, respectively.^21^ In females the skin elastance also increased with age, becoming softer (Figure 3).^11^, ^12^

Skin viscoelastic properties at the four anatomical sites are associated with leg volume at the malleolus and knee, with the tone decreasing with increasing volume (Figure 4). Only in females was skin tone altered in the proximal region as well. Skin stiffness was also related to volume at the knee site in both sexes; although only in the females was there a relationship between the proximal site and volume (Figure 5). At no sites was the distal skin parameters related to volume.

This is the first study to report the relationship between lower limb volume and skin biomechanical properties measured by the myotonometer. A larger sample population would allow a closer inspection of the relationships between leg volume and body characteristics, as aged‐matched, height‐matched, and weight‐matched participants could then be compared.

## 5 CONCLUSIONS

In adults, there is a positive correlation between body size and leg volume. This relationship is further influenced by age and sex. Leg volume alone is independent of sex but is best predicted by body weight, volume increasing with body weight. Skin biomechanical properties (such as tone, stiffness, and elastance) are influenced by sex and leg volume. Different regions of the leg exhibit different skin properties according to age and sex. Thus, studies on skin and leg volume should be mindful of the participants age and sex, as limb volume changes are accompanied by changes in the skin's biomechanical properties.

## CONFLICT OF INTEREST

The authors report no conflict of interest.
